# Solid-, Solution-, and Gas-state NMR Monitoring of ^13^C-Cellulose Degradation in an Anaerobic Microbial Ecosystem

**DOI:** 10.3390/molecules18089021

**Published:** 2013-07-29

**Authors:** Akira Yamazawa, Tomohiro Iikura, Amiu Shino, Yasuhiro Date, Jun Kikuchi

**Affiliations:** 1 Research Planning and Management Group, Kajima Technical Research Institute, KAJIMA Corporation, 2-19-1 Tobitakyu, Chofu, Tokyo 182-0036, Japan; E-Mail: akira@kajima.com; 2 Graduate School of Medical Life Science, Yokohama City University, 1-7-29 Suehirocho, Tsurumi-ku, Yokohama, Kanagawa 230-0045, Japan; E-Mails: iikura@tsurumi.yokohama-cu.ac.jp (T.I.); yasuhiro.date@riken.jp (Y.D.); 3 RIKEN Center for Sustainable Resource Science, 1-7-22 Suehirocho, Tsurumi-ku, Yokohama, Kanagawa 230-0045, Japan; E-Mail: amiu.shino@riken.jp; 4 Graduate School of Bioagricultural Sciences, Nagoya University, 1 Furo-cho, Chikusa-ku, Nagoya, Aichi 464-0810, Japan; 5 Biomass Engineering Program, RIKEN Research Cluster for Innovation, 2-1 Hirosawa, Wako 351-0198, Japan

**Keywords:** solid-state NMR, solution-state NMR, gas-state NMR, anaerobic digestion, bacterial cellulose

## Abstract

Anaerobic digestion of biomacromolecules in various microbial ecosystems is influenced by the variations in types, qualities, and quantities of chemical components. Nuclear magnetic resonance (NMR) spectroscopy is a powerful tool for characterizing the degradation of solids to gases in anaerobic digestion processes. Here we describe a characterization strategy using NMR spectroscopy for targeting the input solid insoluble biomass, catabolized soluble metabolites, and produced gases. ^13^C-labeled cellulose produced by *Gluconacetobacter xylinus* was added as a substrate to stirred tank reactors and gradually degraded for 120 h. The time-course variations in structural heterogeneity of cellulose catabolism were determined using solid-state NMR, and soluble metabolites produced by cellulose degradation were monitored using solution-state NMR. In particular, cooperative changes between the solid NMR signal and ^13^C-^13^C/^13^C-^12^C isotopomers in the microbial degradation of ^13^C-cellulose were revealed by a correlation heat map. The triple phase NMR measurements demonstrated that cellulose was anaerobically degraded, fermented, and converted to methane gas from organic acids such as acetic acid and butyric acid.

## 1. Introduction

Anaerobic metabolism in complex microbial ecosystems degrades highly polymerized biomass into biogas through certain short-chain fatty acids [1]. It represents one of the most significant anaerobic events on Earth [2] and has received increasing attention as a means to safely produce energy from renewable feedstocks such as industrial organic wastes [3]. Therefore, anaerobic digestion is an indispensable metabolic process for generating organic matter both in Nature and for industrial purposes.

In anaerobic digestion, it is difficult to evaluate and elucidate the metabolic dynamics in the characterization of the degradation processes for refractory polymeric macromolecules such as cellulose. This is due to the complexity of polymeric macromolecules with supermolecular structures and their sequential metabolism resulting from diverse and extensive interactions and competition in microbial ecosystems. For example, natural cellulose, the most abundant polymeric macromolecule on Earth, is a partially crystalline polymer of 1→4-linked β-D-glucose units, and the supermolecular structure of cellulose polymers influences its physical properties and reactivity in synthetic and biological reactions [4]. Natural cellulose exists as crystalline types Iα and Iβ as well as in an amorphous form. In natural cellulose type I fibers, cellulose chains aggregate and form fibrils that are deposited in the cell wall during biosynthesis [5]. These complex structures make it difficult to monitor their anaerobic digestion.

To address the challenge of the structural heterogeneity of polymeric biomacromolecules, solid-state nuclear magnetic resonance (NMR) spectroscopy provides a powerful tool for characterizing the structure and dynamics of biomacromolecules [6,7,8]. For example, it enables observations of crystalline and amorphous structures of cellulose, although not all carbon atoms such as C2, C3, and C5 that overlap in 1-dimensional ^13^C-NMR spectra can be identified. In solid-state NMR, cross-polarization (CP)/magic angle spinning (MAS) is frequently used to analyze the supermolecular structures of cellulose in order to characterize the differences between crystalline and amorphous forms [9,10,11,12].

Moreover, microbial communities can generate acetic acid and butyric acid as metabolites by anaerobic digestion of polymeric macromolecules. Solution-state NMR, NMR-based metabolomics, or metabonomics in combination with multivariate statistical analysis are powerful tools for evaluating metabolic dynamics in microbial ecosystems and have been extensively used to investigate a wide range of biological systems in diverse environments [13,14,15,16,17]. Furthermore, because the low natural abundance (1.11%) of ^13^C contributes to the significantly lower sensitivity of ^13^C-NMR spectroscopy, stable isotope labeling technology is also a powerful tool. We previously developed an approach to monitor metabolic dynamics in microbial ecosystems in order to link relationships between microbial communities and their metabolic potential [18]. The final products of anaerobic microbial metabolism are biogases that can be measured using gas chromatography or gas-state NMR [19,20,21,22]. Therefore, the microbial degradation of complex substrates and the metabolic dynamics of the anaerobic digestion of ubiquitous polymeric macromolecules can be effectively assessed using NMR spectroscopy.

Our previous study characterized the anaerobic degradation of glucose, starch, and cellulose in an ecosystem by determining the metabolic reaction pathway [23]; however, the structural characteristics of cellulose were not evaluated because of the lower sensitivity for detecting carbon. To overcome this obstacle, we prepared ^13^C-labeled bacterial cellulose (BC) to monitor the degradation profiles of anaerobic microbial digestion. ^13^C-BC was added to the anaerobic ecosystem, and its degradation was monitored using solid-, solution-, and gas-state NMR spectroscopy.

## 2. Results and Discussion

This present study focused on characterizing anaerobic digestion catalyzed by microbial ecosystems using solid-, solution-, and gas-state NMR combined with stable isotope labeling technology and multivariate statistical analysis. We characterized the anaerobic digestion of BC, which generates biogas from acetic acid and butyric acid precursors.

### 2.1. Solid-State NMR Spectroscopy

**Figure 1:**
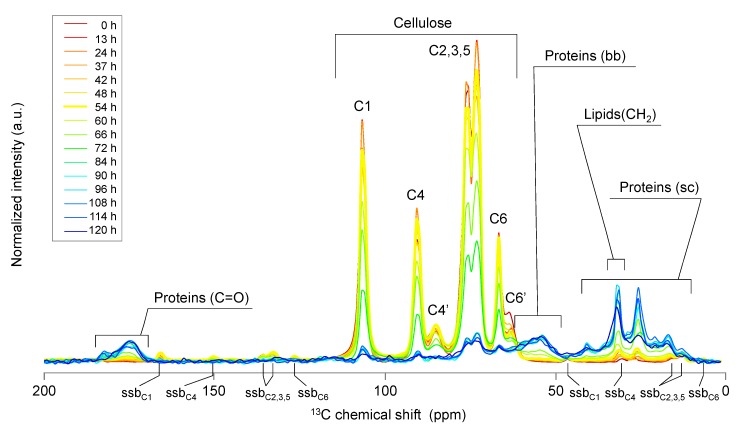
^13^C-CP/MAS-NMR spectra of anaerobic microbial degradation of BC. Sampling time is indicated by the colored lines described in the figure.

Degradation of ^13^C-labeled BC was monitored using solid-state NMR, and the signals generated by proteins and lipids were assigned according to our previous report [24]. We detected each hexose-ring carbon atom (C1–C6) of crystalline and amorphous BC using ^13^C-CP/MAS spectroscopy (Figure 1). The signals specific for BC slightly decreased after its addition to the reactor and remained stable at a low level for 60–84 h after starting the reaction. In contrast, an increase in protein and lipid signals was observed during the same period (Figure 1).

**Figure 2:**
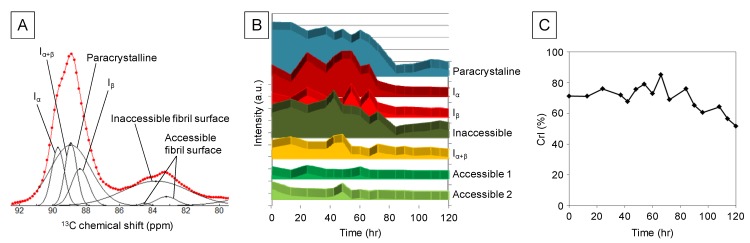
The variations in each BC structure as a function of reaction time. (**A**) Spectral fitting and peak separations of the C4 region of a ^13^C-CP/MAS-NMR spectrum of a representative sample acquired after 13 h. The red dots and the red and black lines represent the raw NMR spectral data, the fitting model, and functions of each allomorph, respectively. (**B**) Intensity fluctuations of each allomorph determined by spectral fitting analysis of the ^13^C-CP/MAS-NMR spectra. (**C**) Time-dependent fluctuation of CrI determined by spectral fitting analysis of ^13^C-CP/MAS-NMR spectra.

Each cellulose peak (for example, the cellulose C4 region) comprises many peaks representing different molecular structures of cellulose, such as cellulose Iα and cellulose Iβ. Therefore, the data were processed to discriminate between the peaks for cellulose Iα, Iβ, Iα+β, paracrystalline, inaccessible, and accessible fibril surfaces (Figure 2A) [25,26,27]. The variations in each BC structure as a function of reaction time are shown in Figure 2B. The signal of the paracrystalline structure slightly decreased at an early stage in the reaction and then immediately decreased (60–84 h). In contrast, cellulose Iα and Iβ signals increased and decreased, respectively, at an early stage and then decreased to the level of the paracrystalline form after 72 h. The signals of the amorphous inaccessible fibril surfaces likely varied proportionally to those of the paracrystalline form and inversely to those of the Iβ form by 72 h. The amorphous structures of accessible fibril surfaces 1 and 2 were detected at low levels, suggesting that the structures were degraded to low-molecular-weight metabolites compared with the other forms of cellulose. Furthermore, the signal intensities of the Iα, Iβ, Iα + β, and inaccessible fibrils decreased after an initial small increase that accompanied the changes in the intact crystalline structures (Figure 2B).

The crystallinity index was calculated as follows (1) [28]:

CrI = Cry/(Cry + Amo)
(1)

where CrI: crystallinity index, Cry: the total area of crystal peaks, Amo: the total area of amorphous peaks.

The crystallinity index was approximately 70%. It fluctuated until 84 h and decreased significantly with the decrease in the cellulose signal (Figure 2C). This result indicates that the degradation rate of amorphous cellulose was faster than that of the crystalline structure. This result is consistent with the small variations in signal intensity of accessible fibril surfaces 1 and 2 (Figure 2B).

### 2.2. Solution-State NMR Spectroscopy

To identify and characterize the metabolism of low-molecular-weight molecules, solution-state ^1^H- and ^13^C-NMR analyses were performed (Figures S1 and S2). The major low-molecular-weight compounds generated from BC were acetic acid, butyric acid, propionic acid, formic acid, and ethanol (Table S1). The ^13^C-NMR chemical shift data were digitized and evaluated, and principal component analysis (PCA) was performed (Figure S3). The PCA score plots show that the metabolic profiles gradually shifted from negative PC1 to positive PC1 directions and from negative PC2 to positive PC2 directions and then returned to negative directions over time. Loading plot analysis revealed that the molecules that contributed to the positive PC1 direction were acetic acid, propionic acid, butyric acid, HCO_3_^−^/CO_3_^2−^, and CO_2_. In contrast, the factors that contributed to the positive PC2 direction or were present in significant amounts in the solutions at 48–72 h were ethanol, acetic acid, and formic acid. These results indicate that these low-molecular-weight metabolites were gradually produced and then accumulated during the first 60 h, and subsequently the concentrations of ethanol and formic acid decreased significantly.

The intensity of the ^13^C-HCO_3_^−^/CO_3_^2−^ signals in solution increased significantly at 40–70 h after the start of reactor operation (Figure S4). There was only a small increase in the intensity of the CO_2_ signal over the same period because the state balance between ions and molecules was dominated by chemical equilibrium [29,30]. The significant increase in ^13^C-HCO_3_^−^/CO_3_^2−^ signals coincided with the decrease in signals of inaccessible fibril surfaces Iα and Iβ, suggesting that the degradation of amorphous and crystalline cellulose occurred simultaneously with the hydrolysis of the polymers.

### 2.3. Relationship between ^13^C-BC Degradation and Production of ^13^C-Labeled Metabolites

**Figure 3:**
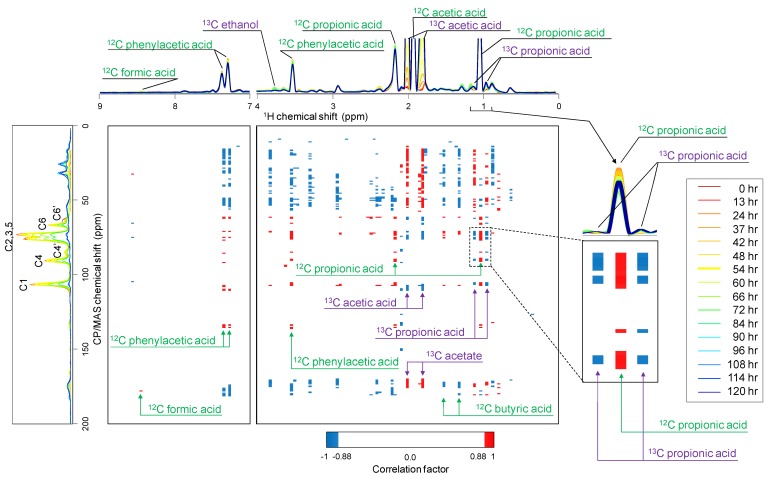
Heat maps comparing solid-state ^13^C-CP/MAS and solution-state ^1^H-NMR spectra. Sampling time is indicated by the colored lines described in the figure. The threshold value of the correlation coefficient was set to a cutoff value of 0.88 as the absolute value in the correlation heat map. Red and blue denote positive and negative correlations, respectively.

The digitized solution-state ^1^H-NMR and ^13^C-NMR chemical shift data were statistically compared with ^13^C-CP/MAS spectra using correlation analysis (Figure 3). The heat map generated by correlation analysis between ^13^C-CP/MAS and solution-state ^1^H-NMR (*i.e.*, the correlation between the structural heterogeneity of cellulose and metabolite heterogeneity) indicated that numerous ^13^C-CP/MAS peaks representing ^13^C-BC correlated positively with ^12^C-^1^H peaks and negatively with ^13^C-labeled low-molecular-weight compounds (Figure 3).

The organic compounds indicated by the ^12^C-^1^H peaks were intrinsically included in the initial anaerobic digestion sludge, and the peak heights decreased as a function of time and cellulose degradation. In contrast, the levels of ^13^C-labeled low-molecular-weight compounds such as acetic acid, ethanol, and propionic acid increased as cellulose was degraded, suggesting that the generation of ^13^C-labeled organic acids was caused by the anaerobic degradation of ^13^C-labeled cellulose. This heat map revealed the relationships of the variations in time course between solid biomass degradation and metabolic dynamics by the microbial communities. In particular, significant cooperative changes between the solid-state NMR signal and ^13^C-^13^C/^13^C-^12^C isotopomers generated by the microbial degradation of ^13^C-BC were revealed by correlation analysis.

### 2.4. Gas-State NMR Spectroscopy

**Figure 4:**
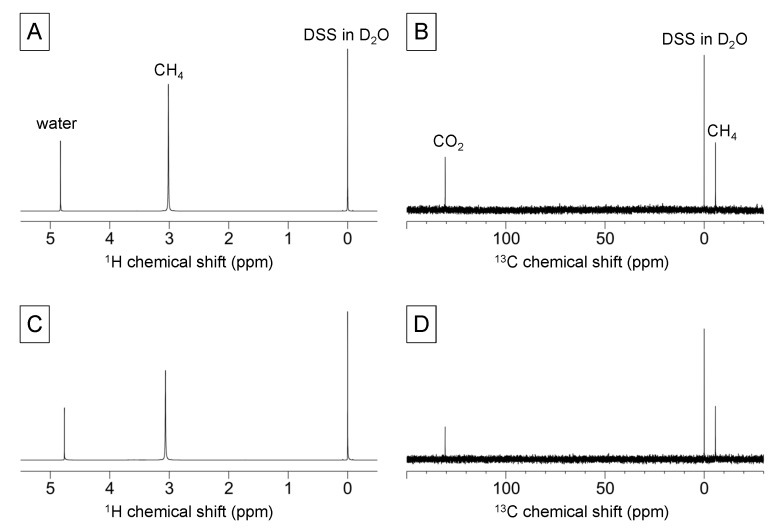
Gas-state ^1^H- and ^13^C-NMR spectra using a double-tube method. (**A**) ^1^H- and (**B**) ^13^C-NMR spectra of a mixture of standard gases (70% CH_4_ and 30% CO_2_). (**C**) ^1^H- and (**D**) ^13^C-NMR spectra of biogas from the anaerobic digestion ecosystem.

The end products of the anaerobic catabolism of BC are CH_4_ and CO_2_ gas. To detect methane and carbon dioxide gas using gas-state NMR spectroscopy, we first prepared a standard mixture of 70% methane and 30% carbon dioxide gas under 4 atmospheres in an NMR tube. The standard mixture was measured using ^1^H- and ^13^C-NMR spectroscopy, and the spectra are displayed in Figure 4A,B. Following this, the gas sample obtained from anaerobic digestion was measured in the same manner, and the ^1^H- and ^13^C-NMR spectra are displayed in Figure 4C,D. The NMR chemical shifts of the standard mixture and the sample were little different between the spectra, suggesting an effect of the partial pressure of each gas [20]. Taken together, this study enabled monitoring of the metabolic dynamics of anaerobic cellulose degradation, short-chain fatty acid production, and methane gas production using solid-, solution-, and gas-state (triple-phase) NMR spectroscopy.

### 2.5. Triple-Phase Monitoring of Anaerobic Digestion Using NMR Spectroscopy

**Figure 5:**
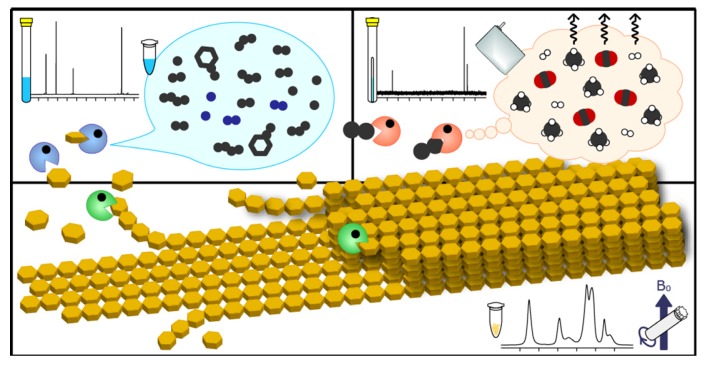
Summary of the thermophilic microbial degradation of BC. The reactions are divided into 3 stages as follows: hydrolysis, acidification, and CH_4_ production. The starting BC substrate was highly crystalline, and its CrI decreased during hydrolysis. Once BC was degraded to glucose, it was immediately catabolized to mainly C_2_ to C_4_ metabolites by acidification (top left). C_2_ acetate was the main source of CH_4_ and CO_2_ (top right).

The results of the present study, acquired using triple-phase NMR spectroscopy, are schematically summarized in Figure 5 and show the entire microbial anaerobic degradation process, starting with a solid biomass substrate composed of crystalline and amorphous BC and resulting in the generation of biogases and low-molecular-weight soluble end products (Figure 5). Through the thermophilic anaerobic digestion of crystalline and amorphous BC, we were able to observe one of the material circulations from solid biomass. Carbon dioxide was initially generated and accompanied by an allomorphic change in the crystalline structure of BC. Following this, the crystalline and amorphous forms of BC were converted to organic acids such as acetic acid and propionic acid. When BC was converted to low-molecular-weight products, the signals of incompletely fermented carbohydrates, for example, glucose, were not detected. We attribute this to the immediate conversion of glucose to organic acids such that the levels of glucose during the process were below the detection limit.

We monitored the entire anaerobic degradation process, starting with a solid biomass substrate and resulting in the generation of gases, using only NMR spectroscopy, which is the only available technique for simultaneously determining triple-phase reactants and products. We believe that this represents a significant advancement in analyzing novel and ready-made bioprocesses such as the methane fermentation process, reported in this manuscript. Moreover, NMR analysis promises to promote further understanding of chemical reactions occurring in microbial community. Such knowledge is indispensable to maximize the efficiency of microbial processes in practical applications.

## 3. Experimental

### 3.1. General

^13^C-labeled BC produced by *G. xylinus* was used as substrate. BC (420 mg) was added to 100 mL anaerobic sludge in 200 mL stirred tank reactors at a constant temperature of 55 °C and gradually incubated for 120 h with microbiota in the anaerobic sludge. The identities and levels of ^13^C-labeled BC degradation products were determined from CP/MAS spectra recorded on a Bruker AV800 spectrometer, and ^1^H- and ^13^C-NMR spectra were recorded on a Bruker AV700 spectrometer. Data were analyzed using multivariate statistical analyses that included PCA and 2D correlation analysis (see below).

### 3.2. Materials

^13^C-labeled BC was produced by cultures of *G. xylinus* supplemented with ^13^C_6_-glucose (^13^C > 99%) purchased from Cambridge Isotope Laboratories (Andover, MA, USA) as described previously [9,10,11,12]. The pellicles of ^13^C-BC produced by *G. xylinus* were converted into powder using an automill machine (Tokken Inc., Chiba, Japan). Powdered ^13^C-BC was incubated in stirred tank reactors at a constant temperature of 55 °C for 120 h with thermophilic anaerobic digestion sludge provided by Kajima Corporation, Japan as described previously [23]. Two mL of samples were collected from the reactors at 0, 13, 24, 37, 42, 48, 54, 60, 66, 72, 84, 96, 108, 114, and 120 h after addition of the ^13^C-BC. The samples were centrifuged (16,500 × *g* for 15 min at room temperature) to separate the pellets from the supernatants for solid- and solution-state NMR measurements.

### 3.3. Solid-State NMR Spectroscopy

Solid-state NMR experiments were performed using an AVANCE-800 spectrometer (Bruker-BioSpin, Billerica, MA, USA) with a Bruker 4-mm MAS triple resonance probe according to previous reports, with slight modifications [9,11]. The rotor was filled with 5–20 mg of precipitate containing ^13^C-BC using polytetrafluoroethylene (PTFE) thread-seal tape (AS ONE Co., Ltd.) as a spacer. The MAS frequency was fixed at 12 kHz. The contact time for CP/MAS and the recycle delay were set to 1 ms and 2 s, respectively. Glycine (specifically, the ^13^C-chemical shift of its carbonyl carbon at 176.03 ppm) was used as the external reference. The acquired spectra were manually phased and baseline-corrected. For peak separation, the line-broadening factor was set to 100 Hz for 0–72 h and 200 Hz for 84–120 h. The NMR data were reduced by subdividing spectra into sequential 0.15 ppm regions between ^13^C-chemical shifts of 50–112 ppm. The binned data were normalized according to the signal-to-noise ratio of each spectrum and the total intensity. Peak separation was performed using Fityk 0.9.8 [31] with the following parameters: “Function type” was “Gaussian”, “fit method” was “Levenberg–Marquardt,” and “add peak” was “manual” [32,33].

### 3.4. Solution-State NMR Spectroscopy

Supernatants suspended in 10% (v/v) deuterium oxide (D_2_O) and 1 mM sodium 2,2-dimethyl-2-silapentane-5-sulfonate (DSS)-d_6_ served as an internal standard. All NMR spectra were recorded using a Bruker AVENCEII-700 spectrometer equipped with an ^1^H inverse triple-resonance cryoprobe with Z-axis gradients. The temperature of NMR samples was maintained at 298 K. For ^1^H NMR spectra, 32768 data points with a spectral width of 12,500 Hz were collected into 256 transients and 1 dummy scan, and residual water signals were suppressed using a Watergate pulse sequence with a recycle delay of 1 s and mixing time of 500 ms. Before Fourier transformation, the free induction decays were multiplied by an exponential window function corresponding to a 2.0-Hz line-broadening factor. For ^13^C-NMR spectra, 66,560 data points with a spectral width of 42,613.637 Hz were collected into 3,072 transients and 2 dummy scans with a recycle delay of 3 s. Before Fourier transformation, the free induction decays were multiplied by an exponential window function corresponding to a 10.0-Hz line-broadening factor. The acquired spectra were manually phased and baseline-corrected. NMR spectra were processed using NMRPipe software [34] and assigned using the SpinAssign program on the PRIMe website [35,36,37].

### 3.5. Gas-State NMR Spectroscopy

Methane and carbon dioxide gas (99.9% pure) were purchased from GL Sciences Inc. (Tokyo, Japan). Biogas was collected in a plastic bag and used for gas-state NMR analysis. We performed NMR analysis using a 5-mm-diameter normal NMR tube (sealed with a rubber plug) with the thin NMR tube (2-mm diameter) that was filled with 100 mM DSS-*d_6_*/D_2_O solution and flame-sealed. The produced biogas was introduced into the 5-mm NMR tube with the thin NMR tube at 4 atmospheres using a gas-tight syringe after evacuation. The thin NMR tube plays important roles in solvent lock, tuning of the shim, and as a semi-internal standard for quantification. Gas-state NMR spectra were measured using a Bruker AVANCEII-700 spectrometer equipped with a ^1^H inverse triple-resonance CryoProbe with Z-axis gradients. The temperature of the NMR samples was maintained at 298 K. For ^1^H-NMR spectra, 65,536 data points with a spectral width of 9,803.922 Hz were collected into 64 transients and 1 dummy scan. For ^13^C-NMR spectra, 65,536 data points with a spectral width of 52083.332 Hz were collected into 256 transients and 4 dummy scans. Before Fourier transformation, the free induction decays were multiplied by an exponential window function corresponding to 0.3-Hz and 1.0-Hz line-broadening factors. The acquired spectra were manually phased and baseline-corrected. NMR spectra were processed using TopSpin 3.1 (Bruker).

### 3.6. Statistical Analysis

^1^H- and ^13^C-solution-state NMR data were reduced by subdividing spectra into sequential 0.04 ppm-designated regions between ^1^H chemical shifts of −0.5 to 9.5 ppm and 0.05 ppm-designated regions between ^13^C-chemical shifts of 8–65 and 113–200 ppm. After exclusion of water resonance, each region was integrated and normalized to the total of the DSS-d_6_ integral regions. ^13^C-solid-state NMR data were reduced by subdividing spectra into sequential 0.8 ppm-designated regions between ^13^C-chemical shifts of 3–200 ppm. The binned data were statistically evaluated by PCA using “R” software according to a previous study [23,38]. A 2D correlation map was calculated as an asymmetric matrix between ^13^C-CP-MAS/NMR data and ^1^H- or ^13^C-solution-state NMR data using Spearman’s rank correlation coefficient according to previous studies [18,23].

## 4. Conclusions

In the present study, we show that anaerobic microbial degradation can be evaluated using a conventional metabolomic analysis strategy applied to plant and animal systems for solution-state NMR in combination with solid- and gas-state NMR using compounds labeled with stable isotopes. We successfully monitored the metabolic dynamics of BC degradation and the production and consumption of short-chain fatty acids and methane using triple-phase NMR spectroscopy, which is the only available method that analyzes the reactions of solid, liquid, and gas phases using a single instrument. Further advancements in NMR analysis will help establish highly efficient microbial processes for producing materials and/or energy. Such microbial processes are indispensable factors to realize the sustainable society.

## Supplementary Materials

Supplementary materials can be accessed at: http://www.mdpi.com/1420-3049/18/8/9021/s1.
