# Supplementary material for: Solid-, Solution-, and Gas-state NMR Monitoring of 13C-Cellulose Degradation in an Anaerobic Microbial Ecosystem
Source: Molecules. 2013 Jul 29;18(8):9021–33. doi: 10.3390/molecules18089021 (PMC6269877; doi:10.3390/molecules18089021)
Supplement: Supplementary file 1 [file molecules-18-09021-s001.pdf]

Supplementary Materials

Figure S1. Solution-state ^{1}H-NMR spectral analysis of BC degradation products generated in an anaerobic digestion ecosystem. Sampling time is represented by colored lines as indicated in the figure.

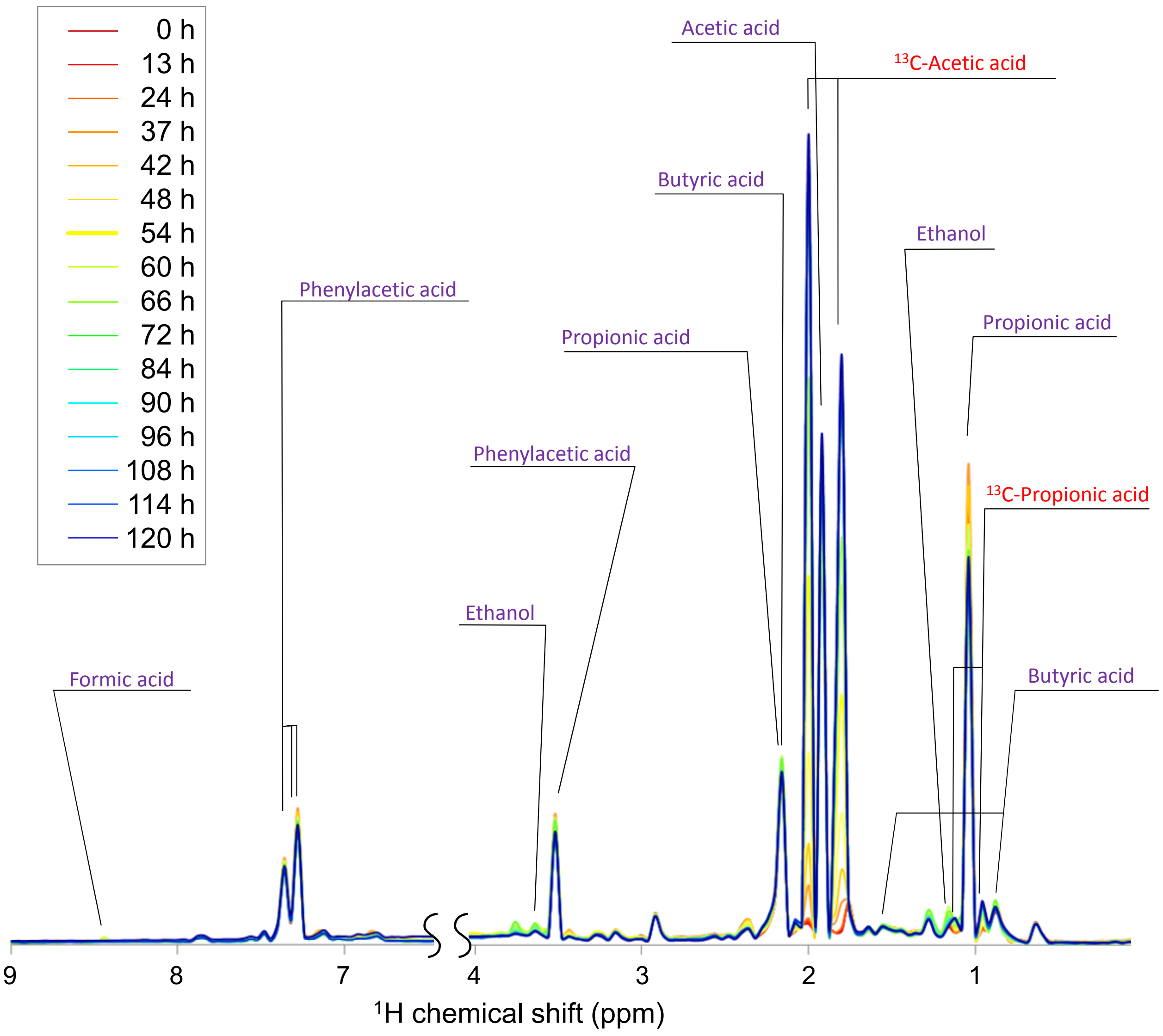

Figure S2. Solution-state ^{13}C-NMR spectral analysis of BC degradation products generated in an anaerobic digestion ecosystem. Sampling time is represented by colored lines as indicated in the figure.

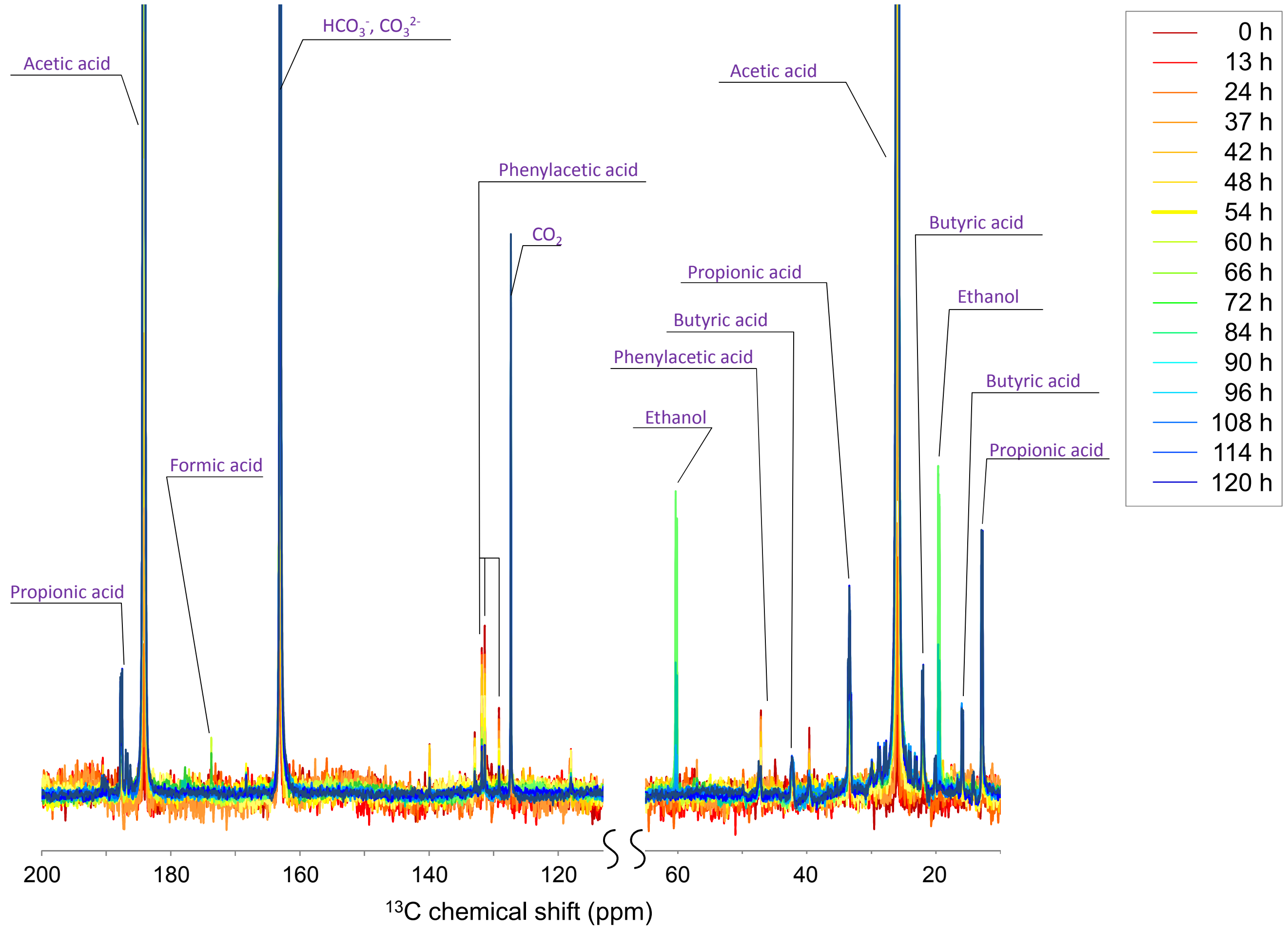

Figure S3. PCA score plot (**A**) and loading plot (**B**) of solution-state ^{13}C-NMR spectra. Sampling time is represented by colored lines as indicated in the figure.

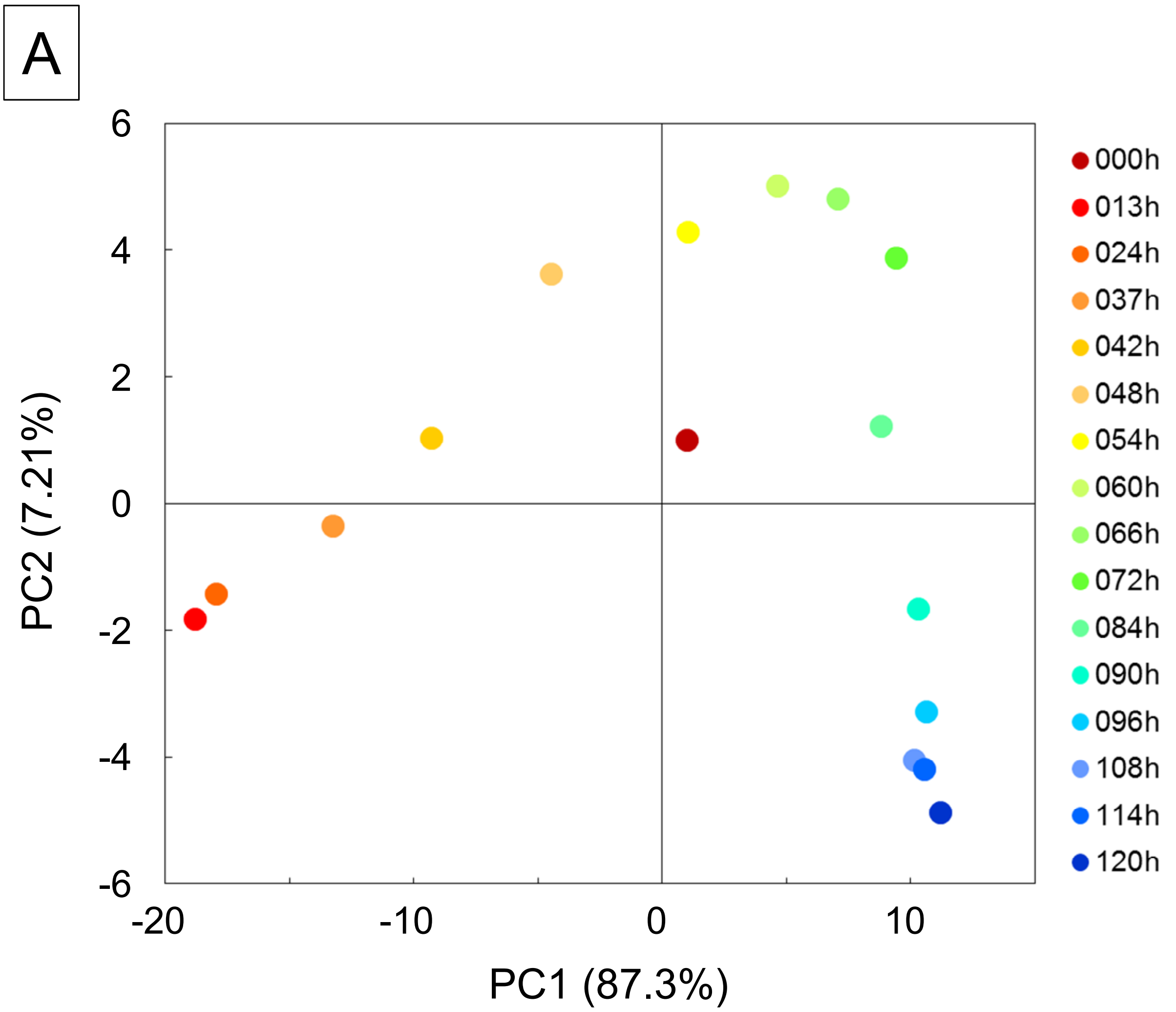

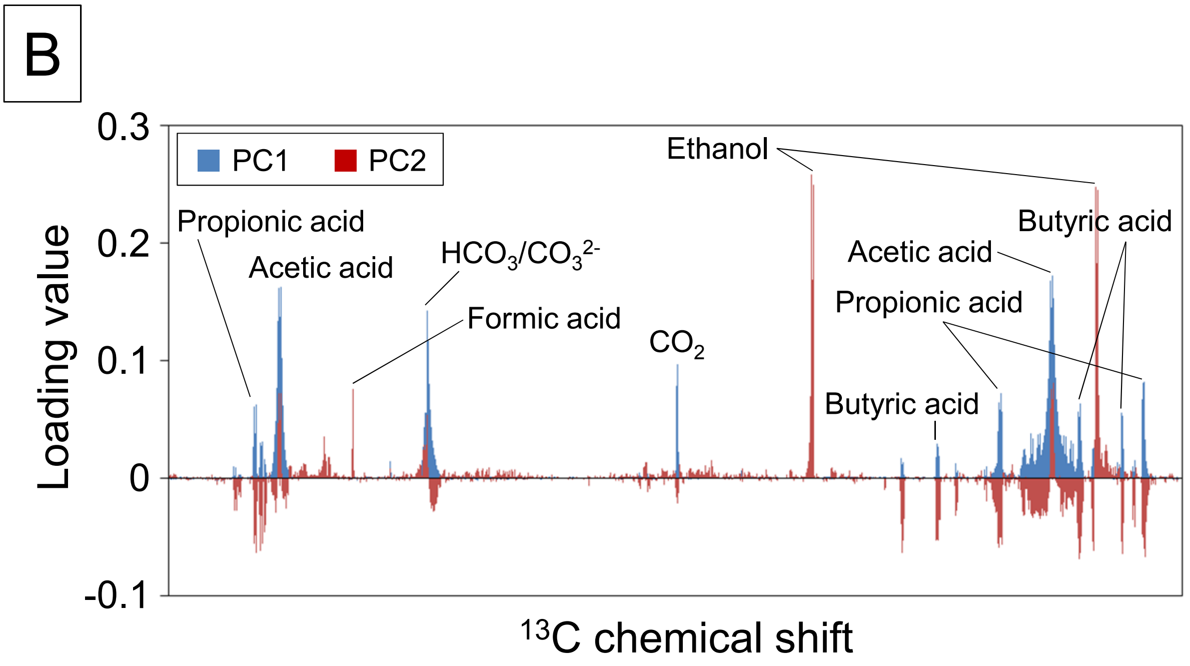

Figure S4. Time course variations of ^{13}C-HCO_3^-/CO_3^{2-} and CO_2 intensities observed in solution-state ^{13}C-NMR spectra of BC degradation profiles.

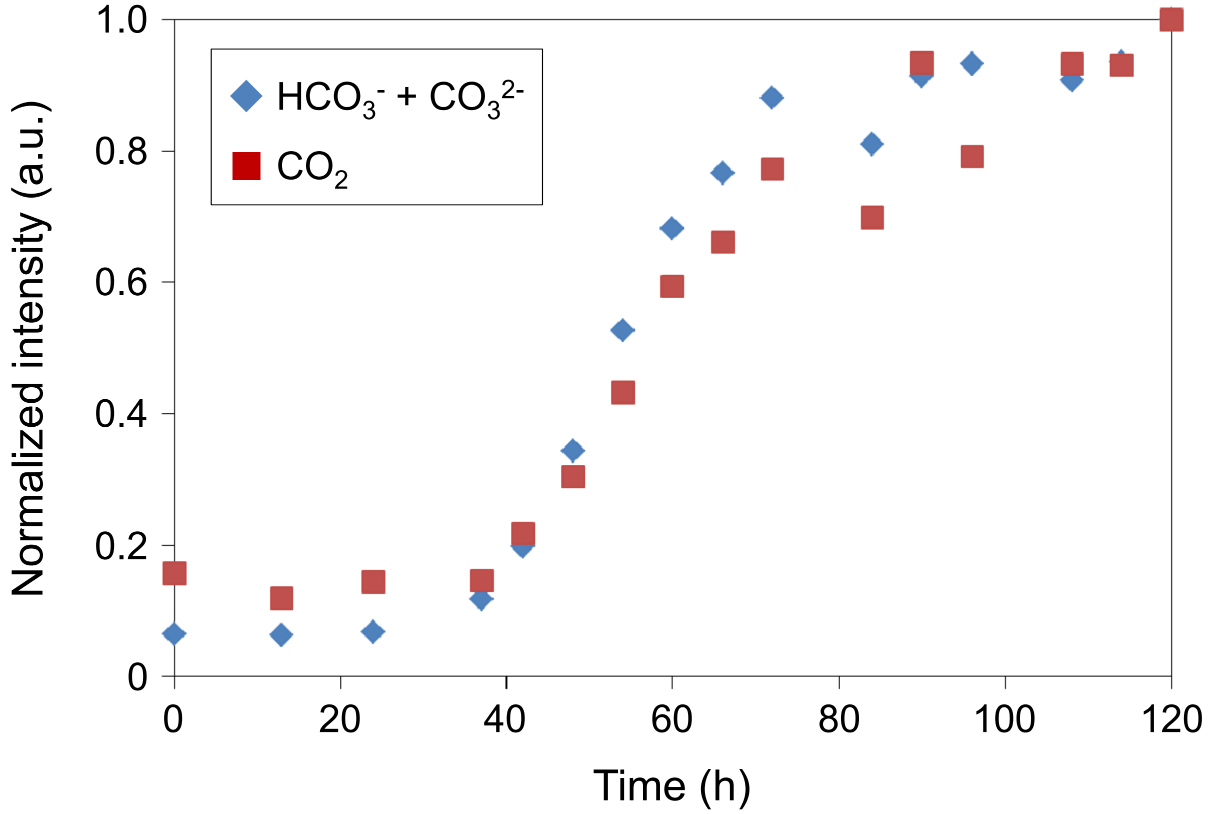

Table S1 Annotated peaks of molecules detected in solid-, solution-, and gas-state NMR spectroscopy.

Substances		^{1}H chemical shift ppm (multiplicity)		^{13}C chemical shift ppm (harf width(Hz) for CP-MAS)	
**Cellulose (solid)**					
	C1			105.18	
	C2, 3, 5			74.68, 71.93	
	C4			89.11	
	C4'			84.28	
	C6			65.38	
	C6'			62.55	
Crystalline Iα	C1			105.01	(179.8)
	C4			89.72	(180.0)
				88.94	(166.9)
	C6			65.26	(204.0)
Crystalline Iβ	C1			105.57	(297.2)
				104.20	(235.0)
	C4			88.39	(242.1)
				88.94	(166.9)
	C6			66.03	(144.6)
				64.62	(245.1)
para-Crystalline C4	C4			88.96	(573.7)
Accessible fibril surface1	C4			84.53	(131.2)
Accessible fibril surface2	C4			83.21	(297.2)
Inaccessible fibril surface	C4			83.83	(940.4)
**Proteins (solid)**					
	sc			9.84-45.53	
	bb			46.84-59.10	
	C=O			166.89-183.01	
**Lipids (solid)**					
	$(CH_2)_n$			30.39	
**Small molecules (liquid)**					
	Butylic acid	0.874	(t)	15.95	
		1.541	(m)	22.08	
		2.137	(t)	42.26	
		-		186.48	
	Propionic acid	1.045	(t)	12.91	
		2.172	(q)	33.41	
		-		187.69	
	Ethanol	1.172	(t)	19.57	
		3.649	(q)	60.23	
	Acetic acid	1.909	(s)	26.0	
		-		184.18	
	Phenylacetic acid	3.528	(s)	47.12	
		7.294	(t)	129.17	
		-		131.85	
		7.373	(t)	131.37	
		-		139.93	
		-		183.80	
	Formic acid	8.443	(s)	173.74	
	HCO_3^-, CO_3^{2-}	-		163.13-163.07	
	CO_2	-		127.34	
**Small molecules (gas)**					
	CO_2	-		130.47	
	CH_4	3.203		-5.53	
